# ﻿First record of the genus *Macrispa* in China, with a redescription of *Macrispa saundersii* Baly, 1859 (Chrysomelidae, Cassidinae, Botryonopini)

**DOI:** 10.3897/zookeys.1252.142681

**Published:** 2025-09-19

**Authors:** Zheng-Zhong Huang, Chao-Fan Zhang, Shi-Xing Leng, Xing-Ke Yang, Si-Qin Ge

**Affiliations:** 1 Institute of Zoology, Chinese Academy of Sciences, Beijing, China Institute of Zoology, Chinese Academy of Sciences Beijing China; 2 Anhui Institute of Optics and Fine Mechanics, Hefei Institutes of Physical Science, Chinese Academy of Sciences, Hefei, China Anhui Institute of Optics and Fine Mechanics, Hefei Institutes of Physical Science, Chinese Academy of Sciences Hefei China; 3 University of Chinese Academy of Sciences, Beijing, China University of Chinese Academy of Sciences Beijing China

**Keywords:** Beetles, faunistics, new record, Oriental region

## Abstract

The genus *Macrispa* Baly, 1859, previously known only from Southeast Asia and south of the Himalayas, has remained unrecorded in China until now. Here, we report the first discovery of *Macrispa saundersii* Baly, 1859 from Xizang Autonomous Region, China, representing a northward range extension for the genus. Through integrative examination of external morphology and male genitalia, we provide a comprehensive redescription of *M. saundersii*. High-resolution stacked imaging was employed to document key taxonomic characters, including elytral punctation patterns, stridulatory files, abdominal ventrite structures, and male genitalia.

## ﻿Introduction

The genus *Macrispa* was established by Baly in 1859, with *M. saundersii* from the Oriental region designated as the type species. Baly noted that this genus differs from *Botryonopa* by having slender, subinerassate antennae and large, convex elytra (Baly 1859). Moreover, the genus was recognized in subsequent catalogs (Gemminger and Harold 1876; Donckier 1899; Weise 1911a, 1911b; Uhmann 1953, 1958). Würmli (1976) synonymized *Macrispa* with *Botryonopa* Guérin-Méneville 1840 and revised the genus *Botryonopa*. However, in Kimoto’s (2005) catalog, the name *Macrispa* was reinstated as a valid genus without any explanation. Staines (2015) also listed *Macrispa* as a valid genus in his catalog, with *M. saundersii* as the sole species under the genus. Concurrently, Sekerka (2015) revalidated the genus *Macrispa* in his revision of the tribe Botryonopini. He provided a key to distinguish between morphologically similar genera within Botryonopini, clearly defining *Macrispa* by the presence of scutellar row of punctures, and irregular punctures on the elytra, which is also the most notable distinction between *Macrispa* and *Botryonopa*. In his study, Sekerka (2015) identified two species under *Macrispa*: *M. ingens* (Gestro, 1903) (syn. *M. perakensis* Maulik, 1929) and *M. saundersii* (Baly, 1859) (syn. *M. krishnalohita* Maulik, 1915).

During an examination of specimens collected in southeastern Tibet in 2023 at the Museum of Hebei University, we discovered a large botryonopine beetle. It was confirmed to belong to the genus *Macrispa* and represents a new record of both genus and species for China. The species is described in detail below.

## ﻿Materials and methods

Specimen depositories:

**BMNH**Natural History Museum, London, UK.

**MHBU** Museum of Hebei University, Baoding, China.

The specimen was softened by soaking in room-temperature distilled water for 24 hours before being removed and positioned. Male genitalia were extracted from the posterior abdomen using fine forceps, placed in a 10% NaOH solution, and heated in a water bath for 5 minutes. Afterward, the genitalia were rinsed thoroughly in distilled water and mounted on white card-paper.

Images were taken using a Canon EOS R5 mirrorless camera paired with a Canon 100mm EF macro lens and a Canon MP-E 65 mm macro lens. Focus stacking was performed using a self-built rail system, and the images were processed with Helicon Focus v. 8.2.2. Final adjustments, including cropping and contrast enhancement, were made in Adobe Photoshop.

The following abbreviations are used throughout the text: **TL** = type locality; **TD** = type depository.

## ﻿Taxonomy

### ﻿Family Chrysomelidae Latrelle, 1802

**Subfamily Cassidinae Gyllenhal, 1813**

**Tribe Botryonopini Chapuis, 1875**

#### ﻿Genus Macrispa

Taxon classificationAnimaliaColeopteraChrysomelidae

Baly, 1859

709AC785-4185-5C9B-A856-A876256D340A

Macrispa Baly, 1859: 90. Chapuis 1875: 294 (redescription); Gemminger and Harold 1876: 3606 (catalog); Baly 1879: 465 (note); Donckier 1899: 559 (catalog); Gestro 1905: 130 (note); Weise 1911a: 40 (catalog), 1911b: 57 (catalog); Maulik 1915: 368 (redescription), 1919: 23 (India species); Uhmann 1954: 39 (catalog), 1958: 160 (catalog); Descarpentries and Villiers 1959: 499 (types); Abdullah and Qureshi 1969: 99 (Bangladesh species, misspelling); Würmli 1975: 10 (genera); Anand 1984: 11 (note); Staines and Staines 1999: 526 (note); Kimoto 2005: 105 (faunal list).
Botryonopa Würmli 1976: 88 (synonymy); Seeno and Wilcox 1982: 162.
Macrispa Sekerka 2015: 718 (stat. restit.).

##### Type species.

*Macrispa saundersii* Baly, 1859, by monotypy; TL unknown, TDBMNH.

##### Diagnosis.

**Figures 1–3.**
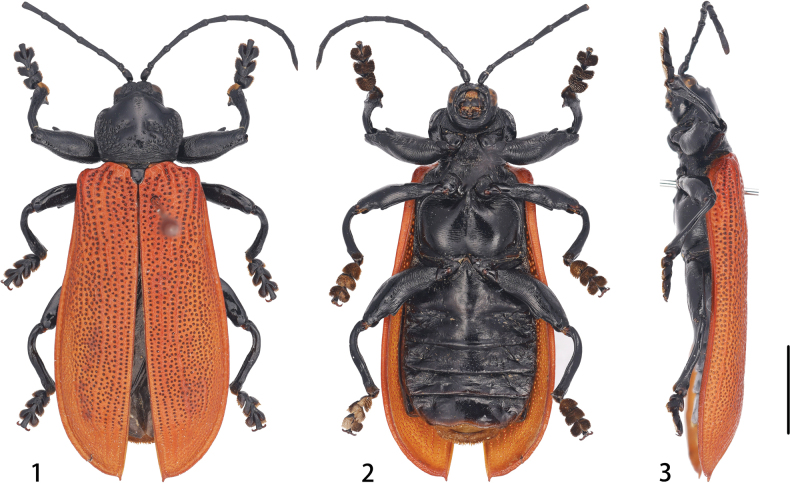
Habitus of *Macrispa saundersii* Baly, 1859 **1** dorsal view **2** ventral view **3** lateral view. Scale bar: 5.0 mm.

The elytra of *Macrispa* are noticeably different from those of *Botryonopa*, as they exhibit predominantly irregular punctation (with only the first two rows being relatively regular) and convex, forming a uniform arch in cross-section (Fig. 1). In contrast, *Botryonopa* has entirely regular punctation and a flattened elytral disc. Additionally, *Macrispa* possesses longer, more slender antennae, which are not tubular like those found in *Botryonopa* (Sekerka 2015).

#### ﻿ Macrispa saundersii

Taxon classificationAnimaliaColeopteraChrysomelidae

Baly, 1859

38877374-2236-529C-8E16-1EB3FC853562

Figs 1–3, 4–9, 10–13

Macrispa saundersii Baly, 1859: 91; TL unknown, TDBMNH.
Botryonopa saundersii (Baly, 1859). Würmli 1976: 88.
Macrispa krishnalohita Maulik, 1915: 369 synonymized by Würmli 1976: 88.

##### Material examined.

CHINA • 1 male; Xizang province, Xiayadong, Kangyang Base; 27°15'22"N, 89°01'10"E; alt. 1832 m; 08 Jul. 2023; Bai Xinglong, Ji Quanyu, and Song Jian leg.; MHBU 20250412.

##### Redescription (based on one male specimen).

Body length: 22.5 mm; body width: 8.0 mm. Body large, flat; elytra orange-red; labrum yellow; remaining parts shiny black.

**Figures 4–9.**
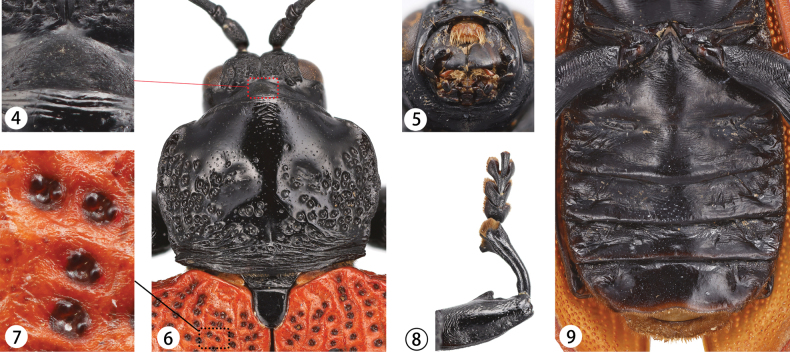
Adult morphology **4** stridulatory files on occiput **5** mouthpart **6** pronotum and scutellum **7** punctures on elytra **8** proleg with single tooth **9** abdomen, venter.

Head narrower than anterior margin of pronotum. Antennae with 11 antennomeres; antennomere 1 robust, cylindrical; antennomere 2 shortest; antennomere 3 longest, slightly longer than antennomere 4; antennomere 4 longer than antennomere 5; antennomeres 6–11 approximately equal in length, with antennomere 11 tapering at the tip. antennomeres 1–4 glossy, antennomeres 5–11 densely covered with short black hairs. Vertex with irregular, coarse punctures, coarser than those on base of pronotum but finer than those on pronotal disc. Compound eyes elongate, oval, finely faceted. Vertex with depressions near compound eyes, which bear coarse punctures and long, yellow hairs. Occiput with triangular stridulatory file area (Fig. 4). Gena smooth, impunctate or only few shallow wrinkles. Short, deep median groove between antennae. Frons smooth, without punctures, with depressions near compound eyes, containing dense yellow hairs. Mouthparts (Fig. 5) hypognathous. Labrum yellow and densely covered with long, yellow hairs along anterior margin. Mandibles robust. Maxillary palps with 4 palpmeres; palpomere 1 shortest, palpomeres 2–4 swollen, palpomere 4 longest with flattened, truncated yellowish tip; palpomeres 2–4 bear scaly surface with several punctures. Labial palps with 3 palpomere; palpomere 1 shortest, palpomere 2 broad, palpomere 3 longest, with yellowish, truncate tip.

Pronotum (Fig. 6) flattened, with weak wrinkles near the anterior margin. Anterior third relatively smooth with fine and weak punctures; coarser irregular punctures present on sides, some punctures confluent. Two depressions present on either side of central area, separated by smoother region with fine punctures. Base of pronotum with strong transverse wrinkles. Sides of pronotum slightly curved and rough, with irregular rounded edges that taper slightly near base. Anterior angles rounded; posterior angles nearly right-angled, neither sharp nor prominent.

Scutellum black, shiny, tongue-shaped, smooth, impunctate.

Elytra longer than abdomen, fairly flat, and broader at base compared to pronotum. Each elytron has prominent humeral region and folded edge extending to apex. Elytra hairless but with coarse punctures, more prominent on disc and sides, weakening toward apex. Each coarse puncture accompanied by two shallow circular protrusions (Fig. 7). Elytra with mesh-like surface with weak ridges. Sides expand toward apex, with weak lateral edges. Exterior apical angle rounded, with small tooth at each sutural angle.

Prosternum with lateral folds mostly smooth with a few coarse punctures. Base of prosternum, like base of pronotum, with strong transverse wrinkles. Procoxal cavities closed, and prosternal process very narrow, with strong wrinkles, strongly arched, expanding significantly toward wide, flat, and nearly straight apex.

Mesosternum T-shaped, with strong wrinkles. Lateral parts flatter with weak punctures.

Metasternum with transverse wrinkles, stronger on sides and weaker at middle. Weak median line present, slightly depressed toward anterior margin. Posterior sides strongly wrinkled, becoming weaker toward apex.

All femora thicker than tibiae, especially profemur. Males have one or two transverse lamellate protrusions on midsection of each femur (Fig. 8). All femora with wrinkles, particularly near base. Tibiae slightly curved, with long groove on ventral side. Apex of tibiae swollen, with short dense bristles, especially on protibia, with lamellate protrusion ventrally. Tarsi 4-4-4; first three tarsomeres flattened, wide, and densely covered with short hairs ventrally. Third tarsomere longer than either first and second tarsomeres. Claws simple.

Abdomen flattened, with five visible sternites; sterna 1 and 2 appear fused medially, remaining sternites clearly separated (Fig. 9). Central part of each sternite with weak wrinkles, with stronger folds and depressions on sides. Last sternum smooth, with wavy edges, a rounded depression in center, and short, dense, yellow hairs.

**Figures 10–13.**
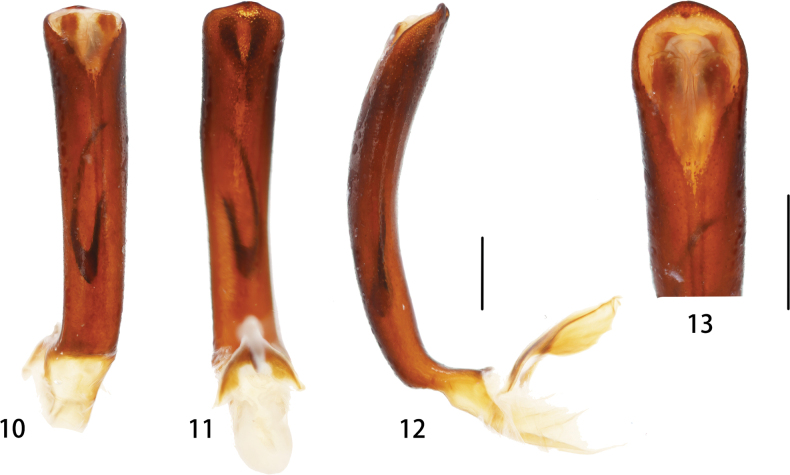
Median lobe of *Macrispa saundersii* Baly, 1859 **10** dorsal view **11** ventral view **12** lateral view **13** apex. Scale bar: 1.0 mm.

Male genitalia (Figs 10–13). Median lobe of aedeagus strongly sclerotized, with parallel sides and slightly expanded base and apex; in lateral view, median lobe of aedeagus slightly curved (Fig. 12). Basal piece unsclerotized. Ventral surface convex proximally, flattened towards apex. In ventral view, widened towards apex, apex rounded (Fig. 13). Tegmen with each arm a little shorter than stem. Stem of tegmen characteristic with a laminate vertical process along middle.

##### Distribution.

China (Xizang, Xiayadong), India, Bhutan, Bangladesh.

## ﻿Discussion

The tropical rainforest in southeastern Tibet, located at latitudes above 29°30'N, is not only the northernmost tropical rainforest in China but also in the world. This region, which is characterized by its steep mountains, deep valleys, and rich biodiversity, provides a suitable environment for many Oriental species, making their presence unsurprising. Due to having only a single specimen and lacking field observations of live individuals, the host plant of this species remains unknown. However, based on its phylogenetic relationship with the genus *Botryonopa* and the documented host plants of *Botryonopa* (Chaboo 2007), it is presumed that this species may be associated with plants from the family Arecaceae (palms), as several species of *Botryonopa* have been recorded on Arecaceae. However, we note that one species of *Botryonopa* has also been associated with Poaceae, indicating that host plant associations in this genus may be broader. In the future, we hope to collect more *Macrispa* specimens from southeastern Tibet to observe their life cycle and fill knowledge gaps. Additionally, molecular techniques are needed to assess the phylogenetic relationship between this genus and *Botryonopa*, as well as its taxonomic position within the subfamily Cassidinae, tribe Botryonopini.

## Supplementary Material

XML Treatment for Macrispa

XML Treatment for Macrispa saundersii

## ﻿Additional information

### Author ORCIDs

Zheng-Zhong Huang https://orcid.org/0000-0002-0085-6218

Chao-Fan Zhang https://orcid.org/0000-0002-5306-2577

Shi-Xing Leng https://orcid.org/0009-0008-4401-9914

Xing-Ke Yang https://orcid.org/0000-0002-0870-4657

Si-Qin Ge https://orcid.org/0000-0001-5924-3400

### Data availability

All of the data that support the findings of this study are available in the main text.
